# Improvement of neuropsychological and cognitive functions in older adults through transcranial vibroacoustic stimulation: a double blind, randomized, comparative trial

**DOI:** 10.3389/fnagi.2025.1526088

**Published:** 2025-08-08

**Authors:** Jang-Han Bae, Jae-Young Ha, Jai Jun Choung, Min-Woo Cho, Byung-In Oh, Kun Ho Lee, Young Chul Youn, SangYun Kim, Dong-Keun Song, Chang-Ho Shin

**Affiliations:** ^1^Digital Health Research Division, Korea Institute of Oriental Medicine, Daejeon, Republic of Korea; ^2^Aging Convergence Research Center, Korea Research Institute of Bioscience and Biotechnology, Daejeon, Republic of Korea; ^3^AriBio Co., Ltd., Seongnam-si, Republic of Korea; ^4^Jangsoo Industry Co., Ltd., Seoul, Republic of Korea; ^5^Gwangju Alzheimer’s Disease and Related Dementias (GARD) Cohort Research Center, Chosun University, Gwangju, Republic of Korea; ^6^Department of Biomedical Science, Chosun University, Gwangju, Republic of Korea; ^7^Department of Neurology, Chung-Ang University College of Medicine, Seoul, Republic of Korea; ^8^Department of Neurology, Seoul National University College of Medicine and Seoul National University Bundang Hospital, Seongnam-si, Gyeonggi-do, Republic of Korea; ^9^Department of AI Convergence Biomedical Engineering, Dongguk University, Goyang-si, Republic of Korea

**Keywords:** transcranial vibroacoustic stimulation, electroencephalography, event-related potential, neuropsychological function, cognitive function

## Abstract

**Introduction:**

Cognitive impairments are critical global public health issues. Recent research has focused on developing non-invasive methods for cognitive enhancement with the potential to slow cognitive decline. This study aimed to explore the effects of transcranial vibroacoustic stimulation (tVAS) on neuropsychological and cognitive functions in older adults.

**Methods:**

This double-blind, randomization, comparative trial applied tVAS at frequencies of 20 or 40 Hz for 30 min per day over an 8-week period using a novel tVAS device. Neuropsychological assessments, saliva cortisol levels, electroencephalography (EEG), and event-related potentials (ERP) were evaluated before and after the intervention.

**Results:**

Following the tVAS intervention, total scores on the consortium to establish a registry for Alzheimer’s disease-Korean version (CERAD-K) 1 and 2 indicated a significant overall improvement in cognitive function in both the 20 and 40 Hz tVAS groups (*p* < 0.01 and *p* < 0.05, respectively). Center for epidemiologic studies depression scale (CES-D) scores after 40 Hz tVAS showed a significant reduction in depressive symptoms compared to baseline (*p* = 0.045), while no significant differences were observed in the 20 Hz group. Individual-level analyses showed that 40 Hz tVAS, but not 20 Hz, increased power across all EEG frequency bands and enhanced N100 and P200 ERP component amplitudes. Group-level comparisons revealed pronounced differences in the gamma band and a significant increase in P200 amplitude in the 40 Hz group.

**Discussion:**

Individual-level EEG and ERP analyses suggest that 40 Hz tVAS enhances neural plasticity, and early-stage sensory processing efficiency. Group-level comparisons further support the successful induction of gamma entrainment, potentially promoting brain network synchronization and increased sensitivity to high-frequency auditory stimuli. While the 40 Hz tVAS intervention demonstrated potential cognitive and affective benefits with favorable safety characteristics, larger-scale studies are needed to confirm its clinical applicability.

**Clinical trial registration:**

https://cris.nih.go.kr/, identifier registration KCT0010055.

## 1 Introduction

As the global population continues to age, characterized by increasing life expectancy and a growing elderly demographic, the prevalence of cognitive impairments has emerged as a critical public health issue (Alzheimer’s Association, 2013; Prince et al., 2015; Knopman et al., 2021) Recently, several monoclonal antibodies targeting amyloid beta have been approved as the first disease-modifying therapies for Alzheimer’s disease (AD) (Sims et al., 2023; van Dyck et al., 2023). However, only modest effects are achieved with these treatments, and serious side effects, such as amyloid-related imaging abnormalities (ARIA), are frequently observed. Therefore, there remains a paucity of effective non-invasive measures to combat this disorder.

To address these challenges, recent research has focused on developing non-invasive methods for cognitive enhancement with the potential to slow cognitive decline. These approaches include transcranial direct-current stimulation (tDCS), transcutaneous electrical nerve stimulation (TENS), transcranial magnetic stimulation (TMS), and transcranial ultrasound stimulation (TUS) (Bhattacharya et al., 2022). However, these techniques face limitations such as low precision, inability to reach deeper brain structures, and the presence of side effects (Bartel et al., 2017; Blanco-Duque et al., 2024). Moreover, the cognitive improvements achieved are often short-lived. For instance, tDCS and TMS have limited penetration into deeper brain tissue, TENS is primarily utilized for pain management, and research on the application of TUS for cognitive enhancement is still in its early stages (Chen et al., 2019).

In an additional line of investigation, various auditory and music-based interventions, such as neurologic music therapy (NMT), music-supported rehabilitation (MSR), rhythmic auditory stimulation (RAS), acoustic coordinated reset (ACR), and physio-acoustic therapy (PAT) have been developed to enhance neural plasticity and improve motor and cognitive functions in patients with neurological disorders (Rochester et al., 2009; Miyazaki et al., 2013; Jirakittayakorn and Wongsawat, 2017). These methods utilize rhythm, frequency-based, and acoustic stimuli to target specific neural pathways, demonstrating potential in the rehabilitation of motor functions, neuro-activation, and sensory processing. However, despite their therapeutic promise, these approaches are constrained by the lack of standardized protocols, insufficient large-scale clinical trials, and limited studies on long-term efficacy.

Rhythmic auditory stimuli may enhance cognitive functions related to learning and memory, and tactile vibrations may similarly elicit neural responses, suggesting their potential as complementary sensory interventions (Campbell et al., 2021; Monteiro et al., 2021). To address the aforementioned limitations of auditory-based interventions, alternative approach such as vibroacoustic therapy (VAT), which utilize low-frequency vibrations and sound waves to deliver widespread neural stimulation throughout the body, are currently being explored (Punkanen and Ala-Ruona, 2012; Bartel et al., 2017). VAT primarily aims to promote relaxation, reduce stress, and alleviate pain while also demonstrating potential benefits for cognitive function. It has proven particularly effective in enhancing cognitive function in neurodegenerative conditions such as AD, indicating that vibrotactile and auditory stimulation can improve cognitive clarity and memory by driving oscillatory power (Ribary et al., 1991). Furthermore, VAT can influence neural coherence and oscillatory activity, suggesting its role in modulating neural oscillations (Bartel et al., 2017).

The more recent and emerging technique of transcranial vibroacoustic stimulation (tVAS) represents a novel approach, applying vibrational stimulation directly to the brain through the skull (Kong et al., 2025). This method offers targeted localization and appropriate stimulation depth, potentially enhancing cognitive function through improved neural activity and plasticity. By targeting the frontal and temporal lobe areas closely associated with memory and identified as being most responsive to vibratory tactile stimuli on the head (Myles and Kalb, 2009; de Jesus Oliveira et al., 2016), low-frequency tVAS may provide effective stimulus delivery. Additionally, since the hippocampus is situated at the edge of the temporal lobe, targeting this region could further enhance cognitive function (Anand and Dhikav, 2012). Previous studies comparing the effects of transcranial vibration stimulation (TVS) and hand vibration stimulation using resting-state functional MRI analysis have demonstrated that the effects of TVS cannot be solely attributed to peripheral sensory stimulation. Rather, these findings underscore the importance of direct brain stimulation, suggesting that TVS exerts its influence through direct modulation of neural activity rather than via indirect somatosensory pathways (Kong et al., 2025).

In contrast to traditional electro- or magnetic stimulation methods, tVAS offers a novel approach by leveraging the mechanical forces generated by vibrations, which may be a crucial factor in its effectiveness. Mechanotransduction, the process through which cells convert mechanical stimuli into electrochemical activity, involves voltage-gated ion channels that facilitate rapid electrical signal propagation in excitable cells such as neurons (Zaszczynska et al., 2020). The activation of mechanosensitive ion channels, including Piezo1, is essential for cognitive function, as these channels modulate microglial activity and enhance their ability to clear amyloid-beta plaques, which are associated with cognitive decline in AD (Chu et al., 2023; Hu et al., 2023).

The specific frequency utilized in tVAS is also a critical factor, as memory processes occur within distinct frequency ranges that influence cognitive functions (Kong et al., 2025). Low frequency vibrations can be delivered through various therapeutic modalities, with tactile stimulation shown to induce relaxation responses beneficial for individuals with dementia (Campbell et al., 2021). In particular, gamma-frequency waves are effective for observing the integrative processes of human cognition, allowing for the visualization of neural network synchronization and binding that cannot be detected with other functional brain imaging methods (Kong et al., 2025). Cognitive deficits in AD are associated with reduced gamma power around 40 Hz, suggesting that vibrotactile and auditory stimulation at this frequency may enhance cognitive function by boosting oscillatory power (Blanco-Duque et al., 2024). Gamma rhythms around 40 Hz are vital for memory and attention, and tVAS’s focus on this frequency promotes neural entrainment, enhancing neural coherence and information processing, potentially leading to improved cognitive clarity and memory (Bartel et al., 2017; Sharpe et al., 2020). Furthermore, 40 Hz sub-sonic vibrations (SSVs) are effective in promoting neuronal differentiation of human umbilical cord mesenchymal stem cells by influencing cell growth inhibition, morphological changes, and critical signaling pathways (Cho et al., 2012).

To investigate whether the effects are frequency-specific beyond 40 Hz, studies utilizing other frequencies, such as 20 Hz, have indicated that the 20 Hz frequency may not effectively engage the mechanisms involved in microglial activation and Aβ clearance (Bartel et al., 2017; Singer et al., 2018). However, since these studies primarily focused on neural activity stimulation and the reduction of amyloid beta using light flicker, further research is needed to explore the effects and mechanisms related to the response of mechano-sensitive ion channels based on 20 Hz tVAS.

To evaluate the extent of cognitive improvement, studies utilizing bio-signals such as electroencephalography (EEG) and event-related potentials (ERP) have been extensively explored (Katmah et al., 2021). EEG analysis techniques allow for direct and non-invasive measurement of brain activity, offering valuable insights. For instance, EEG studies recording brain responses have demonstrated that different visual and auditory stimulation paradigms can elicit the highest gamma activity or most widespread effects (Jirakittayakorn and Wongsawat, 2017; Sharpe et al., 2020; Park et al., 2022). Additionally, VAT can influence various EEG frequency bands, including alpha, beta, and theta waves, contributing to relaxation, stress reduction, and cognitive enhancement. ERPs, which reflect electrical brain activity in response to specific events or stimuli, provide a consistent and reliable measure of neural responses to auditory or visual stimuli (Sheen et al., 2024). Despite its potential, there is a paucity of studies utilizing EEG and ERP in the context of tVAS.

This study aims to explore the effects of an 8-week tVAS intervention using a new tVAS device on neuropsychological functions in older adults through a pilot study. Changes in neuropsychological characteristics, stress levels, and EEG and ERP measures before and after the tVAS intervention were assessed, providing insights into its effectiveness.

## 2 Materials and methods

### 2.1 Participants and experimental design

**FIGURE 1:**
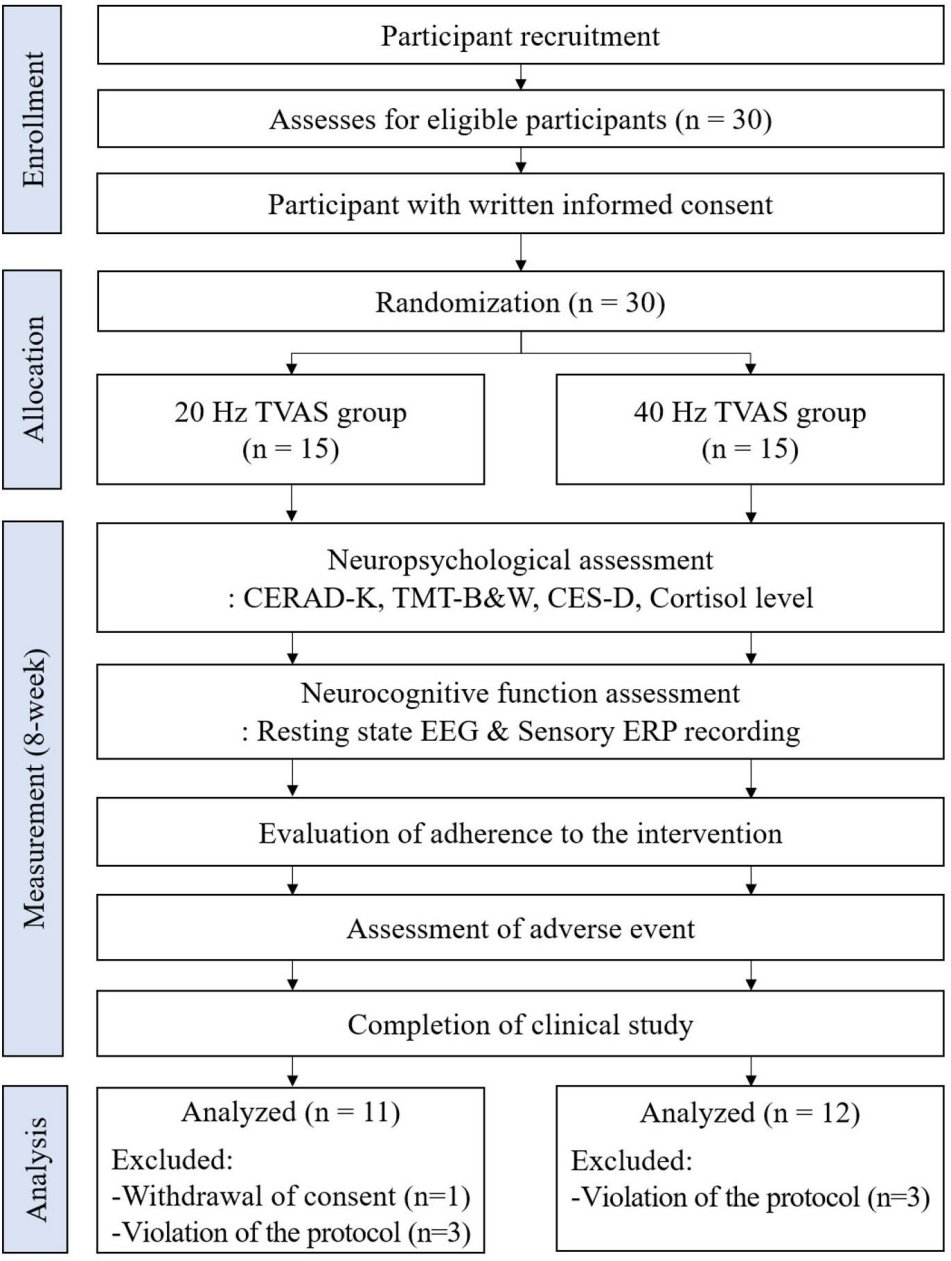
The experimental workflow, in accordance with the consolidated standards of reporting trials (CONSORT).

Participants aged 55 to 85 years of both genders were recruited from Gwangju Bitgoeul Senior Health Town, South Korea. The study protocol was approved by the Institutional Review Board of the Asian Dementia Foundation, South Korea (Approval No. 2024-01-01). This trial was registered with the Korean Clinical Trials Registry, known as the Clinical Research Information Service (CRIS), under the identifier number KCT0010055. The first patient was enrolled on February 13, 2024. The trial adhered to the principles of the Declaration of Helsinki. Recruitment was conducted through public advertisements, and all participants provided written informed consent after receiving a comprehensive explanation of the study procedures. The study was designed as a double-blind, randomized, comparative trial with 30 participants. Random assignment to intervention groups was achieved using a computerized block randomization method, with a block size of 4 and a 1:1 allocation ratio, ensuring balanced group distribution. The random allocation sequence was generated by an independent statistician who was not involved in participant enrollment or the administration of interventions. The randomization sequence and allocation were concealed from all study participants, research staff, and investigators until the study was completed. The sample size was determined considering the exploratory nature of this pilot study, aiming to assess the potential effects of the tVAS intervention on neuropsychological and cognitive functions and to gather foundational data for future study designs. Participants were randomly assigned to one of two groups: the 40 Hz stimulation group (40 Hz tVAS group, *n* = 15) or the 20 Hz stimulation group (20 Hz tVAS group, *n* = 15). The experimental workflow, in accordance with the consolidated standards of reporting trials (CONSORT), is shown in Figure 1.

The eligibility criteria for participants were as follows: (1) individuals aged between 55 and 85 who were capable of participating in the study; (2) those without severe hearing, visual, or speech impairments that could interfere with the administration or completion of neuropsychological assessments; (3) individuals with no history of alcohol dependence or other substance abuse, no history of stroke, and no evidence of central nervous system diseases or injury. Only participants who voluntarily agreed to participate and signed the informed consent form were included. Individuals with cognitive impairments were excluded, and only healthy participants were enrolled.

The exclusion criteria for participants were predefined as follows: (1) individuals with acute illnesses requiring treatment; (2) individuals with a history of central nervous system disorders; (3) individuals on long-term psychiatric medications; (4) individuals diagnosed with visual or auditory impairments; (5) cancer patients who have undergone treatment within the past three years prior to screening; (6) individuals who have participated in other research studies within the past three months; (7) individuals who have experienced a stroke within the past 24 months; and (8) individuals deemed unsuitable for the study by the investigator for any other reason. Participants were discontinued from the clinical trial if, after enrollment, they met any of the following conditions: experiencing device usage issues, such as skin allergies; meeting exclusion criteria identified post-screening; inability to complete follow-ups; or if continuation was deemed inappropriate by the clinical investigator.

Participants attended two sessions: an initial baseline assessment and a follow-up after 8 weeks. Cognitive function was assessed using the consortium to establish a registry for Alzheimer’s disease-Korean version (CERAD-K), executive function using the Trail Making Test–Black & White Version (TMT-B&W), and depression levels using the Center for Epidemiologic Studies Depression Scale (CES-D), both before and after the intervention. The CERAD-K is a neuropsychological assessment tool developed for the early diagnosis of Alzheimer’s disease and the evaluation of cognitive function, with overall scores derived from various cognitive domains: attention, memory, language, visuospatial function, and executive function (Na et al., 2023). The CERAD total score I (TS I) was calculated by summing the scores of six tests (verbal fluency, Boston Naming Test, word list memory, word list recall, word list recognition, and constructional praxis), while CERAD total score II (TS II) was derived by adding the score of the constructional recall test to TS I, with both TS I and TS II used to assess global cognitive function (Seo et al., 2010; Han et al., 2014; Byeon et al., 2021). The TMT-B&W is a neuropsychological test designed to assess visual attention, processing speed, cognitive flexibility, and set-shifting abilities by requiring participants to connect numbers and letters in sequential order, distinguished by black and white colors (Jang et al., 2016). It consists of two subsets: TMT-B&W, part A (TMT-B&W-A), and part B (TMT-B&W-B). The CES-D is a self-report questionnaire designed to screen for depression and assess the severity of depressive symptoms.

Additionally, stress levels were assessed by measuring cortisol levels from saliva samples collected before and after the intervention. Saliva samples were obtained using Salivette^®^ Cortisol tubes (no additives) from SARSTEDT (Schmidt et al., 2021). Sample collection was conducted at 3:00 PM, with participants instructed to refrain from food intake, beverage consumption, and oral hygiene activities for 30 min prior to collection (Shirtcliff et al., 2015). The collected samples were immediately stored at −80°C and later transferred to the analysis facility for further processing. The samples were subjected to up to five freeze-thaw cycles during the analysis. Cortisol levels in saliva were measured using a Cortisol Saliva ELISA kit (Batch No. RE52611, IBL International, Hamburg, Germany) based on a competitive enzyme-linked immunosorbent assay (ELISA) (Schmidt et al., 2021). Absorbance was measured at 450 nm, with all samples analyzed in duplicate. The coefficient of variation (CV) was generally within 5%, and concentrations were determined using a standard curve constructed on the same plate (Shirtcliff et al., 2015). The inter-assay precision of the kit ranged from 3.2% to 6.1%, with the standard curve covering a concentration range of 0.005 μg/dL ∼ 2.209 μg/dL. Cross-reactivity with related steroid compounds was minimal, ranging from 0.001% to 0.01%.

Subsequently, EEG and ERP signals were recorded to assess neurocognitive function. Detailed procedures are described in Section “2.3 EEG and ERP recording.”

### 2.2 Intervention using the tVAS device

The device used in this study, referred to as tVAS, is a head-worn vibroacoustic device designed with ergonomic principles to maximize user comfort and efficiency. The device features an adjustable length band and silicone pads for enhanced comfort during wear. It is certified under the KC (Korea Certification) with the number R-R-ABo-BVD-W1. The tVAS device incorporates an ultra-small acoustic vibration module that delivers low-frequency vibroacoustic stimuli specifically designed for application to the frontal and temporal lobes. The frontal and temporal lobes, closely linked to memory and highly responsive to vibratory tactile stimuli, were selected for stimulation; the inclusion of the temporal lobe also facilitates effective stimulus delivery to the adjacent hippocampus (Myles and Kalb, 2009; Anand and Dhikav, 2012; de Jesus Oliveira et al., 2016).

**FIGURE 2:**
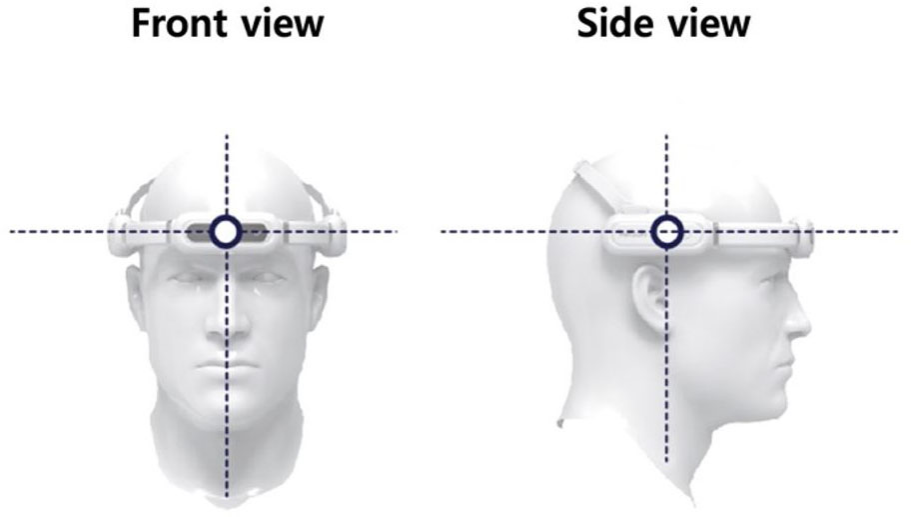
The tVAS (transcranial vibroacoustic stimulation) device.

The tVAS device, as shown in Figure 2, was worn on the head, with adjustments made to ensure it remained securely in place. During use, the silicone pads, designed for skin contact, were positioned to touch the forehead (targeting the frontal lobe) and both sides of the head (targeting the temporal lobes). If the device began to slip, the adjustable band was tightened to prevent any displacement.

The intervention fundamentally employed a mechanical stimulus with a frequency of 40 Hz, as research has demonstrated that low-frequency vibrations at this frequency can promote the differentiation of neurons and support the repair and regeneration of damaged neural tissues (Cho et al., 2012). Additionally, to explore whether 20 Hz stimulation would produce different effects compared to 40 Hz or function as a control frequency with no effects, we also included the option to administer 20 Hz stimulation in order to examine potential placebo effects. 20 and 40 Hz sound sources were delivered at approximately 30 dB through bone conduction to stimulate the auditory nerves. This method was designed to utilize vibratory tactile stimulation to activate mechanosensitive ion channels, which may offer neuroprotective effects, thereby alleviating symptoms associated with memory and cognitive decline. Both intervention groups used devices that were identical in size, color, and operational sounds, differing only in frequency settings, ensuring that participants could not distinguish between the intervention types.

Participants were instructed to use the tVAS device twice daily (excluding weekends) for 15 min per session. To monitor compliance, self-report logs were provided, and participants were asked to record the dates and times of usage over the 8-week period. These logs were submitted during the second visit, 8 weeks after the intervention began. Additionally, regular check-ins via phone and messages were conducted to verify device usage, ensure log completion, and monitor any adverse reactions.

### 2.3 EEG and ERP recording

EEG and ERP signals were recorded before and after the intervention using the NeuroNicle FX2, a portable device developed by LAXTHA in Daejeon, Republic of Korea. This device has been extensively utilized in hospitals, public institutions, and dementia centers, with its reliability and the accuracy of the EEG measurements well-established in previous research (Choi et al., 2019). Additionally, the device is optimized for simple and efficient measurement, making it highly suitable for home use alongside the tVAS device. For the recordings, two non-invasive monopolar scalp electrodes were positioned on the prefrontal regions, specifically at Fp1 and Fp2, following the international 10/20 electrode system. The reference electrode was placed on the right earlobe. Contact impedances were maintained below 10 kΩ, and data were sampled at a rate of 250 Hz with 15-bit resolution. To ensure high signal quality, digital infinite impulse response Butterworth filters were applied, including a 1st-order high-pass filter at 2.6 Hz, an 8th-order low-pass filter at 43 Hz, and a 2nd-order band-stop filter with a frequency range of 55 to 65 Hz (Doan et al., 2021). Additionally, trained clinical research nurses monitored participants to minimize artifacts from blinking, muscle movements, and external noise. Any segments of the EEG or ERP signals affected by participant movement, drowsiness, or external disturbances were excluded from the analysis.

In this study, EEG signals were recorded with participants in an eye-closed, upright position during spontaneous brain activity to establish background EEG signals in a resting state over 5 min (resting-state EEG). Additionally, eight auditory stimuli at frequencies of 125, 250, 500, 750, 1,500, 2,000, 3,000, and 4,000 Hz were presented over 8 min, with a total of 480 stimuli, to elicit sensory-evoked potentials (sensory ERP). These stimuli were presented in a pseudo-random sequence to prevent consecutive repetition, thereby avoiding sensory adaptation and maintaining response sensitivity (Pérez-González and Malmierca, 2014). The selection of eight stimulus frequencies, spanning both high and low ranges, was aimed at capturing hearing loss patterns typical of aging (Ciorba et al., 2011; Rigters et al., 2016). Each stimulus, delivered through earphones at 70 dB, lasted 50 ms with 1 ms rise and fall times and a 1-s interval between stimuli.

### 2.4 Preprocessing and variables of EEG and ERP

All recorded EEG and ERP signals underwent visual inspection, and data contaminated by eye or muscle noise, as well as unexpected external signals, were excluded from the analysis. Resting-state EEG data from two participants were excluded from the analysis due to poor signal quality, as determined by visual inspection criteria. An EEG and ERP analysis program was developed in MATLAB R2023a using EEGLAB functions.

For the analysis of resting-state EEG, a finite impulse response (FIR) bandpass filter ranging from 1 to 50 Hz was applied to minimize noise in both high- and low-frequency bands. Four-second epochs were extracted, and baseline correction was performed. Epochs exceeding an amplitude of 100 μV or exhibiting high variability compared to the overall variability of all epochs were automatically identified and discarded. Artifact-free 45 epochs (3 min) were randomly selected for quantitative EEG analysis (Bae et al., 2024). The EEG power spectrum density (PSD) was calculated using the Pwelch method with a 2-s Hamming window and 50% overlap to minimize distortions at signal boundaries. Absolute powers were computed for the following five frequency bands: delta (1–4 Hz), theta (4–8 Hz), alpha (8–13 Hz), beta (13–30 Hz), and gamma (30–50 Hz). Relative powers for the delta, theta, alpha, and beta bands were calculated as relative delta, theta, alpha, and beta power, respectively, based on the 1–30 Hz range. Peak power was identified as the maximum spectral power within the frequency range of 6.5–14.5 Hz, and individual alpha peak frequency (IAPF) was measured within 7–14 Hz to identify the center of gravity frequency, minimizing sensitivity to noise (Christie et al., 2017). In addition, average PSD plots for the considered frequency bands were obtained both before and after tVAS stimulation for visual comparison.

For the analysis of sensory ERP, we employed a preprocessing and ERP component extraction method specifically designed for two-channel ERP data from the prefrontal lobe (Bae et al., 2022). This method followed several key steps: filtering, epoching, baseline correction, artifact rejection, random selection, averaging, and ERP component extraction. To minimize noise in both high- and low-frequency bands, a FIR bandpass filter ranging from 0.1 to 30 Hz was applied. Epoching was conducted from −200 to 700 ms relative to stimulus onset, with any epoch exceeding a 100 μV threshold or exhibiting high variability compared to the overall variability of all epochs excluded. Given that each denoised ERP dataset had a different signal-to-noise ratio due to variations in the number of retained epochs, we randomly selected 46 epochs (70% of the maximum number of epochs across the eight types of stimuli) for each of the eight stimulus types, following recommended guidelines (Boudewyn et al., 2018). The final selected epochs were averaged for the left and right ERPs and then combined to create a representative average ERP waveform. For ERP component extraction, the time windows were set to 60–200 ms for the N100 and 180–300 ms for the P200. The latencies and amplitudes of the N100 and P200 components were calculated, with a minimum amplitude threshold of 2 μV, to ensure the reliability of the identified peaks (Bae et al., 2024).

### 2.5 Statistical analyses

The demographic and neuropsychological measures before and after tVAS stimulation for both the 20 and 40 Hz groups were summarized using the per-protocol set. Continuous variables were presented as means and standard deviations through descriptive statistics, while categorical variables were reported as proportions (%) for each category. Data normality was assessed using the Shapiro–Wilk test, and analyses were conducted accordingly, applying either parametric or non-parametric methods. Within-group changes were analyzed using paired *t*-tests, while between-group differences at week 8 were assessed using either independent samples *t*-tests or Mann–Whitney U tests.

A paired sample *t*-test was performed to compare mean differences in resting-state EEG and sensory ERP variables before and after tVAS stimulation in both the 20 and 40 Hz groups, with normality assessed using the Shapiro–Wilk test. When normality assumptions were violated, the Wilcoxon signed-rank test was applied. An independent sample *t*-test was performed to examine the group-level differences between the 20 and 40 Hz groups. The difference scores before and after stimulation were extracted from resting-state EEG and sensory ERP variables for comparison. Normality was assessed using the Lilliefors test. Additionally, Levene’s test was conducted to check the assumption of homogeneity of variances, and when the assumption was not met, Welch’s *t*-test was used. Sensory ERP variables were categorized into three sub-types: low-frequency stimuli (125, 250, 500, and 750 Hz), high-frequency stimuli (1,500, 2,000, 3,000, and 4,000 Hz), and all-frequency stimuli (the combined average of all eight frequencies). Exploratory data analysis was conducted using IBM SPSS Statistics V27, while statistical analysis and data visualization were performed using MATLAB R2023a. Significance thresholds were set at α = 0.05 and 0.01.

## 3 Results

### 3.1 Demographic characteristics

Table 1 presents the demographic characteristics of the study participants. The primary statistical analysis included 11 participants in the 20 Hz stimulation group and 12 participants in the 40 Hz stimulation group. There were no statistically significant differences between the 20 and 40 Hz tVAS groups in terms of sex, age, and education level. Specifically, the distribution of sex was comparable between groups, with 45% male in the 20 Hz tVAS group and 66% male in the 40 Hz tVAS group. The groups also showed similar age distributions and education levels. Of the 30 participants enrolled in the clinical trial, 4 (13.3%) reported mild adverse events, none of which required trial discontinuation. The most common events were intermittent headaches (*n* = 1) and dizziness (*n* = 1), each reported approximately twice during the study period. Additionally, 2 participants reported discomfort around the temples associated with device use. No serious adverse events were observed.

**TABLE 1:** Demographic characteristics.

Variable	20 Hz tVAS (*n* = 11)	40 Hz tVAS (*n* = 12)	*p*-value
Sex (male, %)	5, 45	8, 66	0.414^a^
Age (years)	64.5 ± 8.2	61.5 ± 5.3	0.298^b^
Education level (years)	13.4 ± 3.2	14.2 ± 2.6	0.519^b^

^a^Obtained from a chi-square test.

^b^Obtained from an independent *t*-test.

### 3.2 Effects on neuropsychological assessment

Table 2 presents the neuropsychological assessment scores obtained using the CERAD-K battery in participants before and after the 8-week intervention. In the 20 Hz tVAS group, significant improvements were observed in memory, language, and TS 1 and 2 compared to baseline values. In the 40 Hz tVAS group, attention showed a near-significant improvement (*p* = 0.06), and TS 1 and 2 exhibited significant improvements compared to baseline values. When comparing the 20 Hz and 40 Hz tVAS groups after 8 weeks, only attention domain demonstrated a significant difference between the groups (*p* = 0.015).

**TABLE 2:** Neuropsychological assessment scores using the CERAD-K battery in participants before and after the intervention.

CERAD-K		20 Hz tVAS	40 Hz tVAS	*p*-value
Attention	Baseline	1.69 ± 0.29	1.53 ± 0.37	0.732^a^
	Week 8	1.83 ± 0.28	3.21 ± 1.04	
	Change from baseline	0.13 ± 0.21	1.68 ± 0.80	0.015*^b^
	*p*-value^c^	0.552	0.061	
Memory	Baseline	0.82 ± 0.78	1.40 ± 0.59	0.560^a^
	Week 8	3.41 ± 0.67	2.26 ± 0.87	
	Change from baseline	2.59 ± 0.74	0.87 ± 0.57	0.075^a^
	*p*-value^c^	0.006**	0.156	
Language	Baseline	0.55 ± 0.16	0.86 ± 0.13	0.140^a^
	Week 8	0.87 ± 0.16	0.90 ± 0.11	
	Change from baseline	0.32 ± 0.13	0.04 ± 0.10	0.133^b^
	*p*-value^c^	0.039*	0.707	
Visuospatial Function	Baseline	0.12 ± 0.28	−0.54 ± 0.44	0.437^a^
	Week 8	0.16 ± 0.23	−0.51 ± 0.34	
	Change from baseline	0.04 ± 0.38	0.02 ± 0.51	0.541^b^
	*p*-value^c^	0.917	0.962	
Executive Function	Baseline	1.47 ± 0.52	1.13 ± 0.73	0.752^a^
	Week 8	1.19 ± 0.47	2.84 ± 0.74	
	Change from baseline	−0.31 ± 0.47	1.03 ± 0.75	0.217^a^
	*p*-value^c^	0.545	0.209	
TS 1	Baseline	0.31 ± 0.23	0.11 ± 0.23	0.546^a^
	Week 8	1.12 ± 0.16	0.84 ± 0.29	
	Change from baseline	0.82 ± 0.21	0.74 ± 0.27	0.820^a^
	*p*-value^c^	0.003**	0.018*	
TS 2	Baseline	0.38 ± 0.21	0.23 ± 0.21	0.602^a^
	Week 8	1.17 ± 0.15	0.88 ± 0.25	
	Change from baseline	0.79 ± 0.19	0.65 ± 0.23	0.648^a^
	*p*-value^c^	0.002**	0.017*	

*^a^*Obtained from an independent *t*-test.

*^b^*Obtained from a Mann–Whitney U test.

*^c^*Obtained from a paired *t*-test. TS 1 and TS 2 are presented as z-score.

*p < 0.05;

*s*p < 0.01.

Table 3 presents the results of the TMT-B&W, CES-D, and cortisol levels. The analysis of changes in time-to-completion on the TMT-B&W-A and TMT-B&W-B following the tVAS intervention showed no significant differences when compared to baseline values in both the 20 and 40 Hz tVAS groups. The analysis of changes in CES-D scores after the tVAS intervention indicated that, while the 20 Hz tVAS group showed no significant differences, the 40 Hz tVAS group demonstrated a significant reduction in depressive symptom scores compared to baseline (*p* = 0.045). In terms of saliva cortisol levels, no significant changes were detected in either the 20 or 40 Hz tVAS groups when compared to their respective baseline levels. Additionally, when comparing the 20 and 40 Hz tVAS groups after 8 weeks, no significant differences were observed between the two groups.

**TABLE 3:** Results of the TMT-B&W test, CES-D depression scale represented with scores, and cortisol levels before and after the intervention.

Neuropsychological and stress assessment		20 Hz tVAS	40 Hz tVAS	*p*-value
Time-to-completion on the TMT-B&W-A (s)	Baseline	45.09 ± 4.51	36.25 ± 2.83	
	Week 8	38.73 ± 4.16	33.67 ± 3.05	
	Change from baseline	−6.36 ± 4.36	−2.58 ± 3.32	0.486^a^
	*p*-value^c^	0.581	0.434	
Time-to-completion on the TMT-B&W-B (s)	Baseline	144.45 ± 14.37	112.67 ± 9.25	
	Week 8	137.27 ± 14.67	120.25 ± 9.25	
	Change from baseline	−7.18 ± 4.44	7.58 ± 7.84	0.316^a^
	*p*-value^c^	0.581	0.334	
CES-D	Baseline	12.36 ± 2.24	10.33 ± 1.36	
	Week 8	9.00 ± 2.66	6.92 ± 1.87	
	Change from baseline	−3.36 ± 1.79	−3.42 ± 1.51	0.951^b^
	*p*-value^c^	0.090	0.045*	
Cortisol (μg/dL)	Baseline	0.164 ± 0.034	0.150 ± 0.036	
	Week 8	0.168 ± 0.033	0.134 ± 0.019	
	Change from baseline	0.005 ± 0.037	−0.016 ± 0.032	0.468^b^
	*p*-value^c^	0.906	0.624	

^a^Obtained from an independent *t*-test.

^b^Obtained from a Mann–Whitney U test.

^c^Obtained from a paired *t*-test.

*p < 0.05;

**p < 0.01.

### 3.3 Effects on resting-state EEG and sensory ERP

**FIGURE 3:**
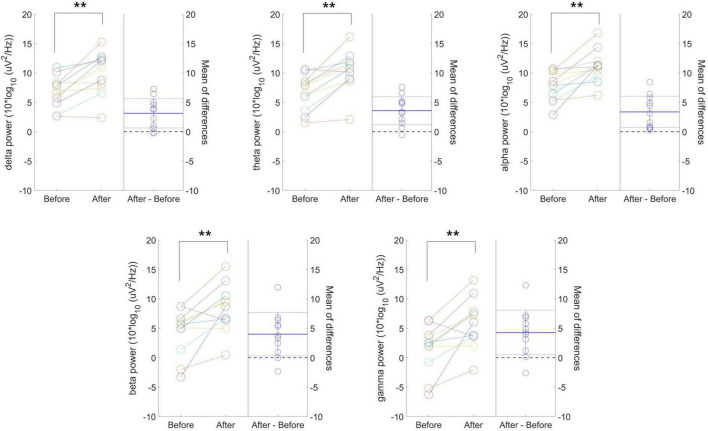
Individual-level changes in delta, theta, alpha, beta, and gamma frequency power values in resting-state EEG for the 40 Hz tVAS group. **p < 0.01.

Figure 3 presents individual-level changes in delta, theta, alpha, beta, and gamma frequency power values in resting-state EEG for the 40 Hz tVAS group. The absolute power values before and after 40 Hz tVAS stimulation for each subject are reported along with the corresponding *p*-values. Additionally, the differences between the two time points are presented alongside the graph, with the mean difference (bold dotted line) and the 95% confidence intervals (thin dotted line). All frequency band powers increased after tVAS stimulation, with statistically significant differences observed across all bands at the *p* < 0.01 level. Notably, greater changes were observed in beta and gamma power.

**FIGURE 4:**
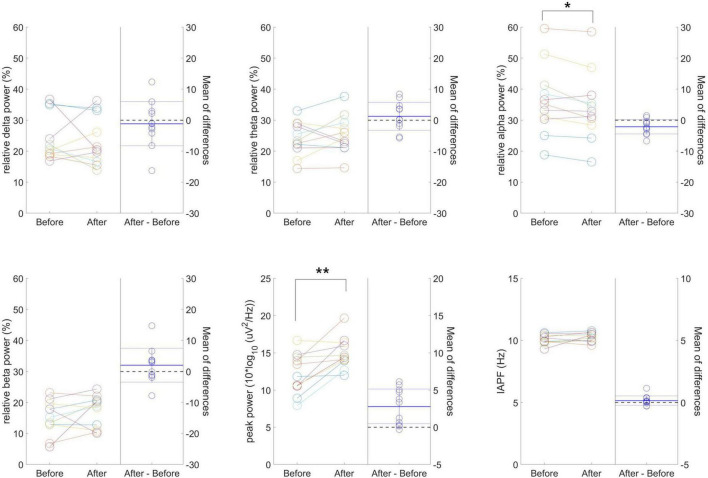
Individual-level changes in relative frequency power and peak-related values in resting-state EEG for the 40 Hz tVAS group. *p < 0.05, **p < 0.01.

Figure 4 presents individual-level changes in relative frequency power and peak-related values (peak power and IAPF) in resting-state EEG for the 40 Hz tVAS group. No significant differences were found in relative delta, theta, and beta power values. However, relative alpha power significantly decreased after 40 Hz tVAS stimulation (*p* < 0.05). Regarding peak-related values, a significant increase in peak power was observed (*p* < 0.01), while no significant differences were detected in IAPF.

The average PSD plots for the considered frequency bands in resting-state EEG before and after 40 Hz tVAS stimulation can be found in the Supplementary Figure 1. The increase in PSD following tVAS stimulation was consistently observed across all frequency bands, including delta, theta, alpha, beta, and gamma.

In the individual-level changes observed in the 20 Hz tVAS group, unlike in the 40 Hz group, no significant differences were found in resting-state EEG variables before and after tVAS stimulation.

**FIGURE 5:**
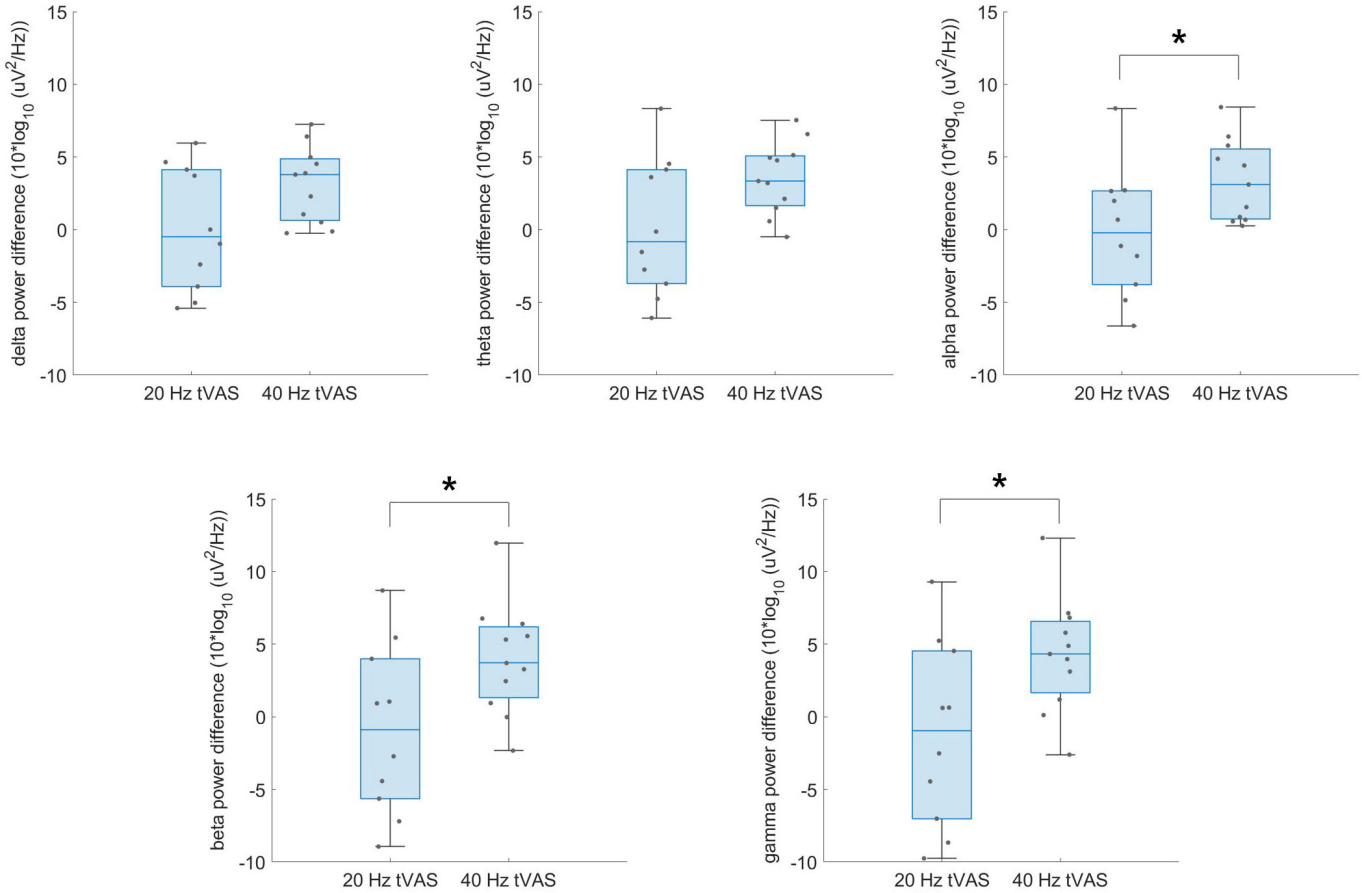
Group-level differences in delta, theta, alpha, beta, and gamma band power difference values between the 20 Hz and 40 Hz groups during resting-state EEG. *p < 0.05.

Figure 5 presents the group-level differences in delta, theta, alpha, beta, and gamma band power difference values between the 20 and 40 Hz groups during resting-state EEG. No significant differences were found in delta and theta power difference, whereas alpha (*p* = 0.039), beta (*p* = 0.034), and gamma (*p* = 0.027) power difference values were significantly higher in the 40 Hz group. Notably, the most significant group difference was observed in the gamma band.

**FIGURE 6:**
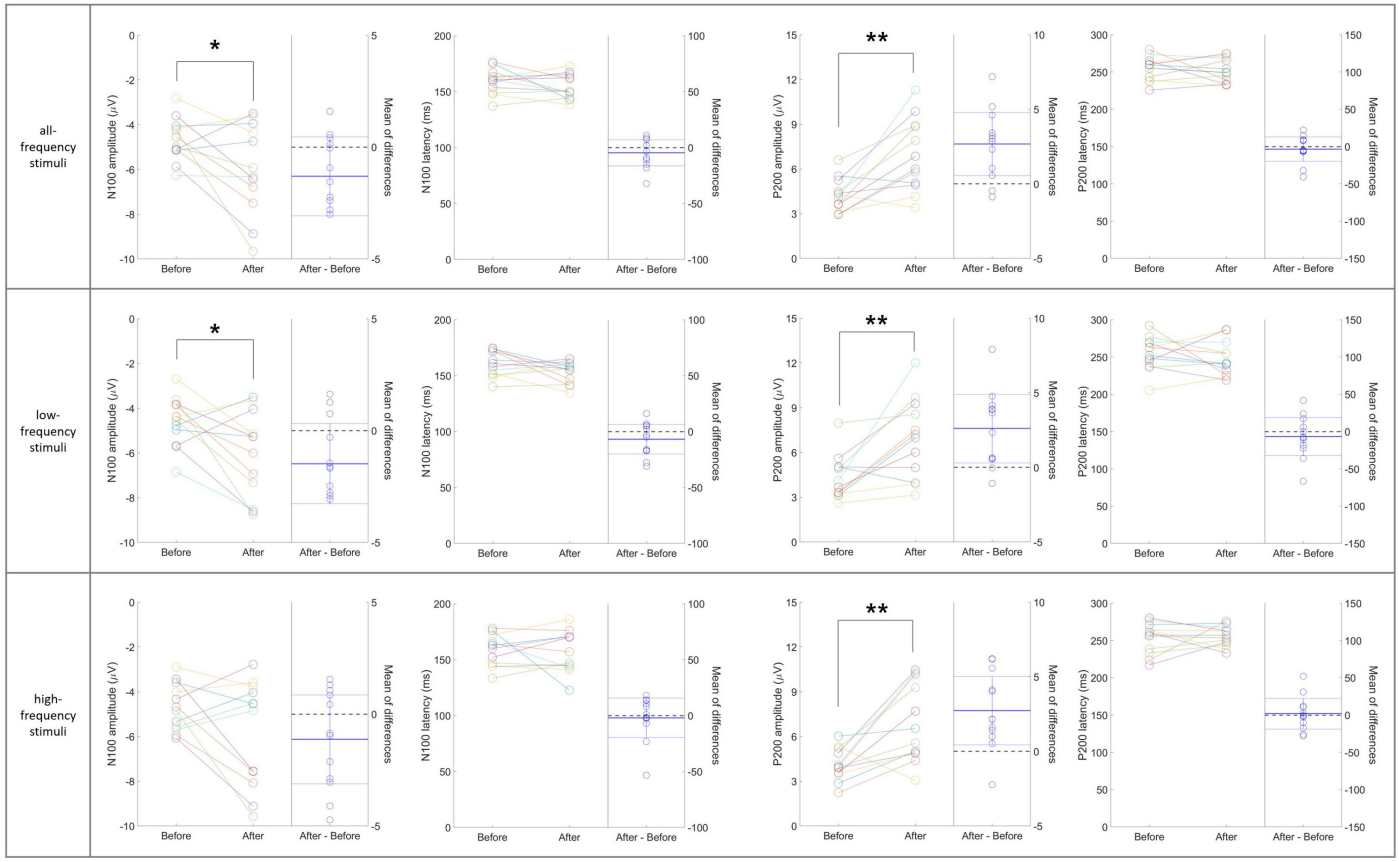
Individual-level changes in the N100 and P200 components of auditory sensory ERPs across the three stimulus sub-types in the 40 Hz tVAS group. *p < 0.05, **p < 0.01.

Figure 6 presents individual-level changes in the N100 and P200 components of auditory sensory ERPs across the three stimulus sub-types in the 40 Hz tVAS group. For the N100 component, the amplitude significantly increased in the negative direction for both the entire frequency range and low frequencies (*p* < 0.05). Amplitude in the high-frequency range also tended to increase in the negative direction, although this was not statistically significant. No significant differences were observed in N100 latency across any frequency range. For the P200 component, amplitude significantly increased across all frequency stimuli ranges (*p* < 0.01). However, no significant differences were observed in P200 latency across any frequency range.

In the individual-level changes observed in the 20 Hz tVAS group, unlike in the 40 Hz group, no significant differences were found in sensory ERP variables before and after tVAS stimulation, except for the N100 latency in response to high-frequency stimuli (*p* < 0.05) (data not shown).

**FIGURE 7:**
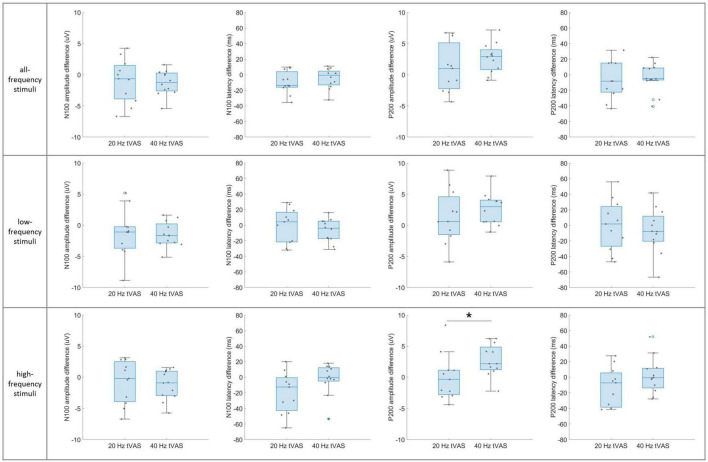
Group-level differences in N100 and P200 component difference values between the 20 Hz and 40 Hz groups in auditory sensory ERP across the three stimulus sub-types. *p < 0.05.

Figure 7 presents the group-level differences in N100 and P200 component difference values between the 20 and 40 Hz groups in auditory sensory ERP across the three stimulus sub-types. A significant difference was observed only in the P200 amplitude difference values in the high-frequency range. In contrast, no significant differences were found in the N100 and P200 component difference values in the all-frequency and low-frequency ranges.

## 4 Discussion

In this study, we analyzed neuropsychological characteristics using a range of assessment tools, stress levels through cortisol measurements, and neurocognitive functions based on EEG and ERP signals before and after the tVAS intervention. The use of a novel multi-sensory stimulation, specifically vibroacoustic stimulation, which directly targets the skull with low-frequency mechanical stimuli at 20 or 40 Hz, represents a distinct approach compared to traditional electrical or magnetic stimulation methods.

Following the tVAS intervention, the CERAD-K TS 1 and 2 indicated an overall improvement in cognitive function in both the 20 and 40 Hz tVAS groups. Notably, after the intervention, a significant difference was observed only in the attention domain between the 20 and 40 Hz groups, suggesting that the 40 Hz tVAS stimulation may have a more positive impact on improving attention function compared to the 20 Hz stimulation. On the other hand, memory and language functions showed significant improvement only in the 20 Hz tVAS group. In the language domain, the baseline value was lower in the 20 Hz group compared to the 40 Hz group (*p* = 0.14), although the post-intervention values were similar between the two groups. This difference in baseline may explain the statistically significant improvement observed only in the 20 Hz group after the intervention. Due to the lack of previous studies examining the effects of 20 Hz tVAS stimulation, well-organized in-depth research with a larger sample size is needed to interpret these findings.

Unlike traditional electrical or magnetic stimulation methods, tVAS uses mechanical forces generated by vibrations, which are converted into electrochemical activity in excitable cells like neurons through a process known as mechanotransduction. This process activates voltage-gated ion channels, facilitating the rapid propagation of electrical signals across neural circuits (Ranade et al., 2015). The activation of mechanosensitive ion channels, such as Piezo1, is crucial in regulating microglial activity and reducing inflammation, providing neuroprotective effects. These mechanisms contribute to enhanced neural plasticity and may aid in the reorganization of disrupted neural circuits (Monteiro et al., 2021; Chu et al., 2023). As a result, tVAS holds potential for improving the function of brain circuits involved in cognitive processes.

In terms of mood assessment, baseline CES-D scores were within the normal range (0–15) for both groups. However, post-intervention CES-D scores following the 40 Hz tVAS intervention showed a significant reduction from baseline (*p* = 0.045), whereas the 20 Hz group exhibited a trend toward reduced depressive symptoms (*p* = 0.090). Although the mechanisms underlying this effect were not directly addressed in this pilot study, the 40 Hz vibroacoustic stimulation may have influenced brain circuits involved in emotional regulation, potentially enhancing the function of regions such as the prefrontal cortex and hippocampus, which are known to play key roles in mood regulation. Future clinical trials investigating 40 Hz tVAS interventions in individuals with depression are warranted. Additionally, it would be valuable to examine whether 40 Hz tVAS affects plasma biomarkers associated with depression, such as brain-derived neurotrophic factor, interleukin-6, and cortisol (Nobis et al., 2020).

Baseline salivary cortisol levels were within the normal range for both groups, indicating that the generally healthy older adults in this study did not exhibit high stress levels. The tVAS intervention did not affect these normal cortisol levels. However, it would be worthwhile to investigate the effect of tVAS in individuals with chronic stress, as tVAS may have a stress-relieving effect by potentially lowering abnormally elevated cortisol levels. Future clinical trials involving patients with depression and chronic stress would help clarify whether tVAS can effectively reduce symptoms associated with depression and stress. Overall, tVAS could become a promising, non-invasive intervention for managing both depression and stress.

Individual-level changes in resting-state EEG revealed significant increases in absolute power across all five frequency bands following 40 Hz tVAS stimulation. While an enhancement in gamma or beta power was anticipated with 40 Hz stimulation, the observed increase across all frequency bands suggests that participants underwent multidimensional neural and psychological changes. This widespread increase in brain activation may indicate improvements in functional connectivity and enhanced neural plasticity (Monteiro et al., 2021). The 40 Hz stimulation could have promoted brain synchronization and improved network efficiency and information processing. Generally, alpha waves are prominent in relaxed or resting states, with an increase in alpha power, unlike other frequency bands, being associated with a reduction in cortical activity. Previous studies have shown that patients with AD or mild cognitive impairment (MCI) tend to exhibit lower alpha power at rest compared to cognitively healthy older adults, and this reduction in alpha power is associated with cognitive decline (Jeong, 2004; Lejko et al., 2020). Therefore, increasing alpha power through tVAS, as demonstrated in this study, could be beneficial for cognitive function (Meghdadi et al., 2021). Additionally, prior studies have reported that patients with depressive disorders display reduced alpha power compared to control groups and that elevated stress levels can similarly lead to decrease in alpha power (Al-shargie et al., 2015). The observed increase in alpha power following stimulation in this study suggests that tVAS may have potential effects in reducing depression or stress. Furthermore, the increase in beta and gamma power reflects enhanced focus, attention, and cognitive function (Herrmann et al., 2004). In contrast, the rise in delta and theta power may indicate relaxation, potentially contributing to reduced stress and greater psychological stability among participants (Vanhollebeke et al., 2022).

The significant decrease in relative alpha power following 40 Hz tVAS stimulation suggests that the increase in alpha power was proportionally less compared to the increases observed in the delta, theta, and beta bands. The increase in delta and theta power, typically associated with relaxation and sleep, implies that the intervention may have promoted a relaxed state in the brain (Vanhollebeke et al., 2022). In contrast, the increase in beta power, linked to cognitive processing and focus, suggests that the intervention stimulated cognitive activity (Vanhollebeke et al., 2022). The relative decrease in alpha power, generally associated with relaxation, indicates that the intervention may have led to a greater degree of cognitive stimulation and activation rather than inducing a state of calmness.

The observed increase in PSD peak power suggests enhanced activation of neural networks or improved synchronization of brain waves within specific frequency bands. The absence of a significant change in IAPF indicates that the peak frequency remained stable, implying that the frequency bands maintained their functional roles following the intervention (Christie et al., 2017). This suggests that while the intervention influenced the functional activation of neural networks, it did not alter the inherent characteristics of the frequency bands.

However, the simultaneous increase in power across all frequency bands warrants careful consideration. This phenomenon may indicate that the brain exhibited a non-specific response to the stimulus. Furthermore, excessive increases in beta and gamma power could result in heightened arousal, tension, or even fatigue. Thus, the power increase across all frequency bands may reflect low intervention efficacy or non-specific brain responses, potentially indicating inefficient brain activation or cognitive overload. Future in-depth studies are needed to explore these possibilities further and to gain a deeper understanding of the underlying mechanisms.

Nevertheless, group-level differences between the 20 and 40 Hz groups during resting-state EEG revealed that the intended intervention in the gamma band was successfully implemented, resulting in gamma entrainment. Significant differences were most pronounced in the gamma band, followed by the beta and alpha bands, which are closest to the gamma band. In contrast, no significant differences were observed in the theta and delta bands, which are more distantly located from the gamma band. This suggests that the tVAS successfully induced gamma oscillations, which could potentially impact sensory processing, attention, and memory (Adaikkan and Tsai, 2020). The question remains as to whether gamma oscillations represent functionally meaningful neural mechanisms or if they are merely epiphenomena (Shin and Moore, 2019). However, aberrant gamma activity has been closely linked to memory disorders, particularly AD, with reports of reduced gamma power and coherence between brain regions in both AD patients and mouse models of AD (Iaccarino et al., 2016; Chu et al., 2023). Furthermore, it has been shown that inducing gamma entrainment can affect gene expression in neurons and microglia (Adaikkan and Tsai, 2020), indicating that tVAS could serve as an intervention that influences overall cognitive function.

Individual-level changes in sensory ERP revealed significant increases in the amplitudes of the N100 and P200 components following 40 Hz tVAS intervention, irrespective of the frequency of the three auditory stimulus sub-types. The N100 component is typically associated with the early stages of auditory sensory processing, reflecting the brain’s detection and initial processing of auditory stimuli. An increase in N100 amplitude suggests that the brain’s ability to detect and respond to auditory stimuli has improved, potentially due to heightened neural excitability or more efficient sensory processing pathways (Tivadar et al., 2021). The P200 component, which is linked to attentional processes, the allocation of cognitive resources to sensory input, and the processing of sensory information influenced by stimulus relevance or expectation, also demonstrated an increase in amplitude (Varlamov et al., 2021). This suggests that the intervention enhanced participants’ attentional focus on auditory stimuli and improved the processing of sensory information following its initial detection. In summary, the increases in both N100 and P200 components following the intervention likely reflect an enhancement in the efficiency of early-stage sensory processing and a broader improvement in the brain’s sensory processing capabilities. This suggests that the brain is in a heightened state of readiness or responsiveness, which makes it more likely to generate stronger ERP components.

Additionally, it was observed that the N100 and P200 amplitudes increased consistently across all three sub-types frequency ranges, irrespective of whether they were low or high frequencies. This generalized increase suggests an overall enhancement in auditory processing capabilities, implying more efficient and responsive sensory processing mechanisms in the brain. The uniform response across a broad range of auditory stimuli may indicate improved neural synchronization, heightened sensory detection, and potentially enhanced cognitive processing.

However, pronounced increases in these ERP components could raise concerns about cognitive overload, where excessive brain stimulation may lead to processing inefficiencies or a state of hyperarousal. This is particularly relevant when increases are seen across all frequency bands, suggesting the brain may not be selectively enhancing processing for specific contexts but instead responding uniformly, which could dilute the specificity and effectiveness of cognitive responses.

Group-level differences between the 20 and 40 Hz groups during sensory ERP revealed a significant increase in P200 amplitude differences in the 40 Hz group, specifically in response to high-frequency auditory stimuli (1,500–4,000 Hz). This suggests that 40 Hz tVAS may enhance sensitivity to high-frequency auditory inputs, potentially through the reorganization of sensory or cognitive neural circuits or by promoting plasticity in the corresponding cortical regions (Monteiro et al., 2021; Tonti et al., 2021).

To further explore the relationship between resting-state EEG and ERP responses, we analyzed the correlation between EEG power across all five frequency bands (delta, theta, alpha, beta, and gamma), which showed meaningful changes throughout the study, and the P200 amplitude from ERP. In the 20 Hz group, the correlation coefficients were *r* = 0.73, 0.84, 0.71, 0.67, and 0.66 for delta, theta, alpha, beta, and gamma power, respectively. In the 40 Hz group, the corresponding values were *r* = 0.66, 0.67, 0.68, 0.60, and 0.57. These results suggest a moderate or higher level of association between resting-state EEG, which reflects the overall neural state, and ERP components such as P200, which reflect cognitive and early sensory processing. It is worth noting that the correlations were slightly higher in the control group. This may be due to the greater variability observed in EEG and ERP change scores in the 20 Hz group, which can mathematically inflate the Pearson correlation coefficient by increasing covariance—even in the absence of a true physiological association. In contrast, the 40 Hz group showed more consistent improvements across participants, likely due to the effects of the intervention. This consistency resulted in less variance in change scores, which can lead to a somewhat lower *r* value despite the presence of a meaningful neurophysiological relationship.

Since these are preliminary findings, further studies with larger sample sizes and diverse perspectives are essential. In addition to EEG, incorporating other neuroimaging techniques such as fMRI or PET, could provide a more comprehensive evaluation of tVAS efficacy. Moreover, long-term clinical studies using a within-subject design, comparing pre-stimulation, stimulation, and post-stimulation phases and varying the duration and timing of the intervention will be necessary to determine the persistence and long-term effects of tVAS.

Interestingly, 20 Hz tVAS interventions were effective in improving CERAD-K TSs and showed a near-significant effect on CES-D scores. However, they did not influence resting-state EEG or sensory ERP signals. In contrast, 40 Hz tVAS interventions were more effective than 20 Hz in reducing depressive symptoms and demonstrated a more positive effect on cognitive function, as indicated not only by improvements in CERAD-K scores but also by objective biomarkers, such as resting-state EEG and sensory ERP.

This study demonstrated the effects of tVAS on neuropsychological and cognitive functions, as well as resting-state EEG and sensory ERP signals. Both 20 and 40 Hz tVAS interventions led to significant improvements in the CERAD-K TS 1 and 2. Notably, the 40 Hz tVAS may have a positive impact on enhancing attention function. Additionally, CES-D scores following the 40 Hz tVAS intervention showed a significant reduction from baseline, while the 20 Hz group exhibited a trend toward improvement. The 40 Hz tVAS also resulted in increased power across all frequency bands, including PSD peak power, and significantly enhanced the amplitudes of the N100 and P200 ERP components. These changes suggest improvements in functional connectivity and enhanced neural plasticity. Group-level differences between the 20 and 40 Hz groups revealed that the intended gamma entrainment was successfully induced, suggesting that 40 Hz stimulation may promote brain synchronization, enhance network efficiency and information processing, and increase sensitivity to high-frequency auditory inputs. Moreover, the stronger ERP components observed after the intervention reflect enhanced efficiency in early-stage sensory processing and a general improvement in the brain’s readiness and responsiveness.

Compared to other non-invasive neuromodulation techniques such as tDCS and TMS, tVAS offers practical advantages including ease of use, user comfort, high safety, good compliance, and fewer side effects, making it well-suited for both clinical and home-based applications. Although tVAS is still in the early stages of research and requires the development of standardized protocols for broader use, it is based on a novel approach that enables targeted localization and appropriate stimulation depth. Through mechanotransduction processes, tVAS may enhance cognitive function more effectively than conventional methods.

This study is the first to investigate the effects of tVAS on neurophysiological and cognitive functions using resting-state EEG, auditory sensory ERP, and various assessment tools in older adults. The simple and user-friendly interface of tVAS has the potential to serve as a valuable tool for enhancing cognitive function and alleviating depressive symptoms in this population. However, further research with larger sample sizes is necessary to fully assess its clinical applicability.

## Data Availability

The raw data supporting the conclusions of this article will be made available by the authors, without undue reservation.

## Supplementary material

Supplementary Figure 1 The average PSD plots in resting-state EEG before and after 40 Hz tVAS.
